# Delayed versus primary closure to minimize risk of surgical-site infection for complicated appendicitis: A secondary analysis of a randomized trial using counterfactual prediction modeling

**DOI:** 10.1017/ice.2023.214

**Published:** 2024-03

**Authors:** Amarit Tansawet, Boonying Siribumrungwong, Suphakarn Techapongsatorn, Pawin Numthavaj, Napaphat Poprom, Gareth J. McKay, John Attia, Ammarin Thakkinstian

**Affiliations:** 1 Department of Surgery, Faculty of Medicine Vajira Hospital, Navamindradhiraj University, Bangkok, Thailand; 2 Department of Clinical Epidemiology and Biostatistics, Faculty of Medicine Ramathibodi Hospital, Mahidol University, Bangkok, Thailand; 3 Department of Surgery, Faculty of Medicine, Thammasat University, Pathum Thani, Thailand; 4 Department of Surgery, Faculty of Medicine Ramathibodi Hospital, Mahidol University, Bangkok, Thailand; 5 Centre for Public Health, School of Medicine, Dentistry and Biomedical Sciences, Queen’s University Belfast, Belfast, United Kingdom; 6 School of Medicine and Public Health, and Hunter Medical Research Institute, University of Newcastle, New Lambton, New South Wales, Australia

## Abstract

**Objective:**

To evaluate the risk of surgical site infection (SSI) following complicated appendectomy in individual patients receiving delayed primary closure (DPC) versus primary closure (PC) after adjustment for individual risk factors.

**Design:**

Secondary analysis of randomized controlled trial (RCT) with prediction model.

**Setting:**

Referral centers across Thailand.

**Participants:**

Adult patients who underwent appendectomy via a lower-right-quadrant abdominal incision due to complicated appendicitis.

**Methods:**

A secondary analysis of a published RCT was performed applying a counterfactual prediction model considering interventions (PC vs DPC) and other significant predictors. A multivariable logistic regression was applied, and a likelihood-ratio test was used to select significant predictors to retain in a final model. Factual versus counterfactual SSI risks for individual patients along with individual treatment effect (iTE) were estimated.

**Results:**

In total, 546 patients (271 PC vs 275 DPC) were included in the analysis. The individualized prediction model consisted of allocated intervention, diabetes, type of complicated appendicitis, fecal contamination, and incision length. The iTE varied between 0.4% and 7% for PC compared to DPC; ∼38.1% of patients would have ≥2.1% lower SSI risk following PC compared to DPC. The greatest risk reduction was identified in diabetes with ruptured appendicitis, fecal contamination, and incision length of 10 cm, where SSI risks were 47.1% and 54.1% for PC and DPC, respectively.

**Conclusions:**

In this secondary analysis, we found that most patients benefited from early PC versus DPC. Findings may be used to inform SSI prevention strategies for patients with complicated appendicitis.

Appendectomy is one of the most common abdominal operations, with an annual incidence rate of 100 to 150 cases per 100,000 person years.^1^ Surgical site infection (SSI) is a potential complication following appendectomy, reported as frequently as 7% across all appendectomies,^2^ and as high as 11.2% in low-income countries.^2^ In addition, SSI has been reported to be as high as 9%–53% for complicated appendicitis (ie, ruptured or gangrenous appendicitis), where surgical incisions become contaminated.^3^ This complication is commonly associated with increased reoperation rates and length of stay,^4,5^ which lead to increased treatment costs.^6^ Therefore, SSI prevention is a primary target for improved surgical-care quality outcomes.

Previous studies have recommended delayed primary wound closure (DPC) to reduce SSI occurrence for contaminated surgical wounds.^7,8^ DPC patients receive open wound dressing for 3–5 days before incision suturing.^9^ However, this procedure tends to increase both associated pain from routine wound wet dressing and may lead to increased treatment costs. Studies have shown that DPC failed to reduce SSI compared to primary wound closure (PC) in complicated appendicitis.^3^ Other studies have shown that PC was associated with lower SSI rates compared to DPC.^10,11^ In the randomized controlled trial (RCT)^12^ conducted by our group, we detected no difference in SSI rates among patients randomized to PC versus DPC.

Although the findings from RCTs are highly ranked in the evidence hierarchy,^13^ they only reflect average treatment efficacy at population level. Individual patients may benefit from one therapy or another, even if the overall RCT findings are null or marginal.^14^ In clinical practice, treatment decisions are always made at an individual level and not a population level. As such, clinical outcomes can be improved by using counterfactual prediction modeling approaches that consider individual patients represented by diverse clinical characteristics.^15^

Although trial data^12^ did not reveal a significant effect of PC versus DPC at a population level, it is possible that some subgroups may benefit from PC strategy. Therefore, we reanalyzed our RCT^12^ data using a counterfactual prediction modeling approach to identify patients that would benefit more from either treatment option to reduce overall SSI.

## Methods

This study adhered to the Transparent Reporting of a Multivariable Prediction Model for Individual Prognosis or Diagnosis (TRIPOD) and the Consolidated Standards of Reporting Trials (CONSORT) reporting guidelines.^16,17^

### Data source

This study was a secondary analysis of a previously reported multicenter RCT^12^ comparing PC with DPC in patients who underwent appendectomy due to complicated appendicitis. Briefly, patients were eligible if provided informed consent and they were aged ≥18 years and had gangrenous or ruptured appendicitis; if they were not pregnant; if they had a body mass index (BMI) <40 kg/m^2^; and if they had no history of the following health conditions: autoimmune diseases, HIV, or end-stage renal/liver diseases. Appendectomy was performed via a lower-right-quadrant abdominal incision. In total, 607 patients were consented over an enrollment period from November 2012 to February 2016. Also, 9 participants were lost to follow-up, leaving 598 for inclusion in the secondary analysis.

The study protocol included standardized antibiotic use, intraoperative wound irrigation, closed suction drain, wound dressing, and pain control. Intravenous antibiotics, mainly third-generation cephalosporins and metronidazole, were prescribed and adjusted according to subsequent bacterial culture and antibiotic sensitivity results. In case of penicillin allergy, ciprofloxacin was administered instead. Antibiotics were switched to oral form after 24–48 hours without fever and continued to complete a 7–10-day course. The RCT was registered with ClinicalTrials.gov (NCT01659983) and received approval from the Ethics Committee of Ramathibodi Hospital, Faculty of Medicine, Mahidol University (no. MURA2012/173).

### Predictor variables

We studied 2 study predictors of interest: type of wound closure (PC and DPC) and type of appendicitis (gangrenous and ruptured). In addition, 17 predictors available from the previous RCT^12^ were also considered including 6 operation-related factors (ie, preoperative antibiotic use, incision length, subcutaneous fat thickness, fecal contamination, pus contamination, and closed suction drain) and 11 patient-related factors: sex, age, body mass index (BMI), smoking, American Society of Anesthesiologists’ (ASA) classification, diabetes, hypertension, symptom duration, fever (body temperature ≥ 37.8°C), white blood cell (WBC) count, and anemia (hematocrit ≤ 30%). These factors were considered in our previous SSI prediction model.^18^ Notably, operation time was not considered because this variable was defined after wound-closure time.

### Outcome of interest

The outcome of interest was defined as superficial SSI within 30 days after surgery, as described by the Center for Disease Control (CDC) criteria,^19^ which entailed only skin or subcutaneous tissue incision, subject to 1 of the following conditions: (1) purulent drainage, (2) organisms isolated from fluid or tissue culture, (3) signs and symptoms of infection, or (4) physician diagnosed SSI. Outcome assessors were not blinded to intervention. Patients were followed up at 1 week and 1 month after operation for SSI assessment.

### Statistical analysis

Of the 19 predictor variables, missing values ranged between 0% and 4.5%, leaving 546 of the 598 patients with complete data for analysis. Intraoperative and patient-related data were described separately by SSI groups using mean and standard deviation (or median and interquartile range where appropriate) for continuous data and percentages for categorical data. We applied bivariate logistic regression to assess associations between each of 17 predictors and SSI. Covariates with a *P* value <.10 in a bivariate analysis were simultaneously evaluated in a multivariate logistic regression model that included both study predictors (wound closure and appendicitis type). Forward stepwise selection by a likelihood-ratio test was undertaken to identify significant predictors (*P* < .05) for retention in the final model.

Model performance was assessed by estimation of the area under the receiver operating characteristic curve (AUROC) and calibration coefficient [ie, a ratio of expected/observed values (E/O ratio)] using 1,000 bootstrap replications. An AUROC close to 1 indicates good discriminative performance, and an E/O ratio close to 1 indicates good calibration.

Finally, the odds of SSI were estimated based on the final prediction model, which included type of wound closure, appendicitis type, and significant predictors retained within the model. An individualized prediction of SSI was estimated as follows: First, for individual patients, odds of SSI based on the actual wound closure received and counterfactual odds of SSI if an alternative closure had been received were estimated. Second, these SSI odds were converted to risk or probability of SSI occurrence.^15,20^ Third, an individual treatment effect (iTE) was estimated by subtracting the PC SSI risk from the DPC SSI risk. Average treatment effect (ATE) and potential outcome means (POMs) were calculated from the average iTEs and SSI risks, respectively. All analyses were performed using Stata version 17 software (StataCorp, College Station, TX). Significance was considered at a *P* value threshold <.05.

## Results

**Figure 1.**
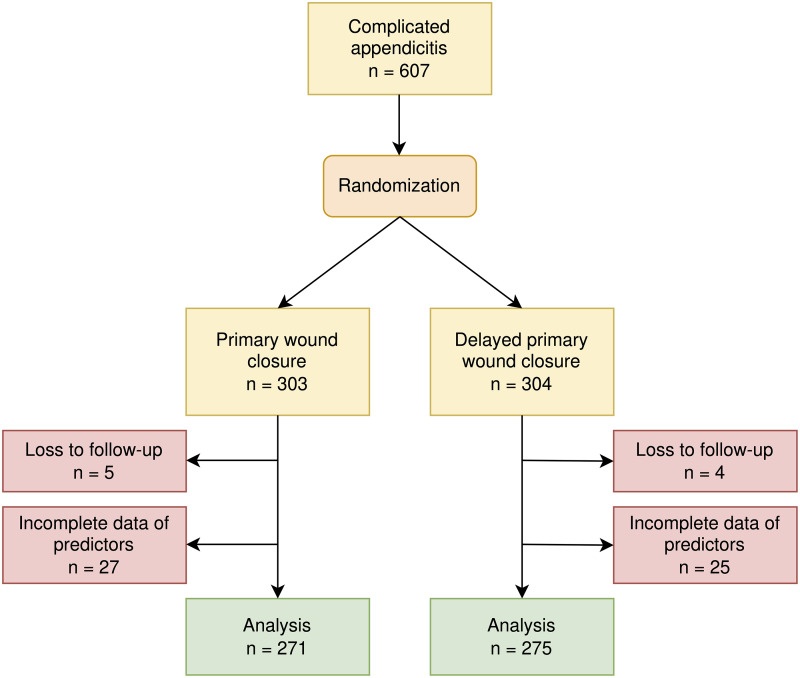
Flow diagram of enrollment and analysis.

Of the 607 participants, 303 and 304 patients were randomized to the PC and DPC groups (Fig. 1). Among them, 271 PC participants and 275 DPC participants had complete covariate data for analysis in the counterfactual prediction model (Fig. 1). The mean age was 45.6 years (SD, 18.2) and 53.5% of patients were male. Most participants were classified as American Society of Anesthesiologists (ASA) class I or II (86.4%). Diabetes prevalence was 9% and hypertension prevalence was 19.8%. Rupture was the main type of complicated appendicitis (76.6%). No differences between PC and DPC for intraoperative and patient-related data were detected (Supplementary Table S1 online). These data were also described by SSI and non-SSI groups (Table 1). SSI rates were 7.4% in the PC group and 10% in the DPC groups. All SSIs were superficial, but 1 patient in the DPC group progressed to organ-space infection. SSIs were treated by open wound dressing and antibiotic use, and no one required reoperation. The most common causal pathogens were *Escherichia coli* (68.8%), followed by *Pseudomonas aeruginosa* (12.5%), *Proteus mirabilis* (6.3%), and *Enterobacter* (6.3%). These pathogens were not statistically different between PC and DPC (*P* = .974). Among *Escherichia coli* infection, 28.1% were extended-spectrum β-lactamase or multidrug-resistant strains (ie, 28.4% for PC and 27.8% for DPC; *P* = .928).

**Table 1.** Patient-Related Summary Characteristics and Intraoperative Factors by Surgical Site Infection Groups

Patient-Related Data	SSI (n=52), No. (%)^a^	Non-SSI (n=546), No. (%)^a^	*P* Value
Age, mean y (SD)	44.4 (16.7)	45.5 (18.2)	.674
**Sex**			
Male	29 (9.1)	291 (90.9)	.733
Female	23 (8.3)	255 (91.7)	
BMI, mean kg/m^2^ (SD)	24.5 (3.3)	23.3 (4.4)	.023
**Smoking**			
Smoker	9 (9.6)	85 (90.4)	.905
Nonsmoker	43 (8.6)	460 (91.5)	
**ASA classification**			
Class I or II	44 (8.6)	470 (91.4)	.622
Class III or IV	8 (10.3)	70 (89.7)	
**Diabetes**			
Diabetes	11 (21.6)	40 (78.4)	.001
No diabetes	41 (7.6)	502 (92.5)	
**Hypertension**			
Hypertension	10 (8.8)	104 (91.2)	.989
No hypertension	42 (8.7)	439 (91.3)	
Symptom duration, median h (IQR)	24 (24, 48)	24 (14, 48)	.055
**Fever**			
Present	38 (11.5)	292 (88.5)	.025
Absent	14 (5.4)	247 (94.6)	
WBC count, mean cells/mm^3^ (SD)	16,658 (4,658)	15,564 (4,998)	.130
Hematocrit, mean % (SD)	41.2 (9.7)	38.8 (6.1)	.084
**Preintraoperative data**			
**Wound closure**			
PC	22 (7.4)	276 (92.6)	.256
DPC	30 (10)	270 (90)	
**Preoperative antibiotic use**			
Yes	51 (9)	517 (91)	.503
No	1 (3.3)	29 (96.7)	
**Type of appendicitis**			
Gangrenous	4 (2.8)	141 (97.2)	.004
Ruptured	48 (10.6)	405 (89.4)	
**Pus contamination**			
Contamination	28 (10.7)	234 (89.3)	.127
No contamination	24 (7.1)	312 (92.9)	
**Fecal contamination**			
Contamination	25 (14.9)	143 (85.1)	.001
No contamination	25 (6.2)	378 (93.8)	
Incision length, cm, mean (SD)	6.9 (2.6)	5.6 (2.2)	<.001
Subcutaneous tissue thickness, median cm (IQR)	3 (2–5)	2.5 (1.5–4.5)	.027
**Draining use**			
Suction drain use	8 (6.8)	109 (93.2)	.413
No suction drain use	44 (9.2)	433 (90.8)	
Operation time, mean min (SD)	66.9 (43.6)	48.2 (41.3)	.002

Note. ASA, American Society of Anesthesiologists; BMI, body mass index; DPC, delayed primary wound closure; IQR, interquartile range; PC, primary wound closure; SD, standard deviation; WBC, white blood cell.

Furthermore, 7 predictor variables were significantly associated with SSI in univariate analyses. A final parsimonious prediction model was based on the 5 predictors retained, including wound closure (PC vs DPC), diabetes versus no diabetes, ruptured versus gangrenous appendicitis, fecal contamination versus no contamination, and continuous incision length (Table 2) according to the following equation:

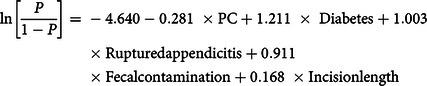




**Table 2.** Predictive Factors of Surgical Site Infection: A Multivariate Logistic Regression Model

Predictor Variable	Multivariate Model	
	Coefficient (95% CI)	*P* Value
**Intervention**		
DPC	0	
PC	−0.281 (−0.907 to 0.345)	.379
**Type of appendicitis**		
Gangrenous	0	
Ruptured	1.003 (−0.076 to 2.082)	.068
**Diabetes**		
No	0	
Yes	1.211 (0.404–2.018)	.003
**Fecal contamination**		
No	0	
Yes	0.911 (0.284–1.539)	.004
Incision length, cm	0.168 (0.061–0.275)	.002

Note. CI, confidence interval; DPC, delayed primary wound closure; PC, primary wound closure.

Of the 5 predictors of SSI, incision length was the strongest, followed by diabetes, fecal contamination, ruptured appendicitis, and wound closure. The model demonstrated good discrimination performance with an AUROC of 0.744 (95% confidence interval [CI], 0.676– 0.812). An internal validation by bootstrapping yielded an AUROC ratio of 0.721 (95% CI, 0.655–0.794) and an E/O ratio of 1.005 (95% CI, 0.750–1.284) (Supplementary Fig. S1 online). The POM_PC_ and POM_DPC_ (ie, average SSI risks) in the PC and DPC groups were 7.7% (95% CI, 7.1%–8.3%) and 9.8% (95% CI, 9.1%–10.5%), respectively. The ATE was −0.021 (95% CI, −0.022 to −0.020); that is, the SSI risk following PC was, on average, 2.1% lower than that observed following DPC.

**Figure 2.**
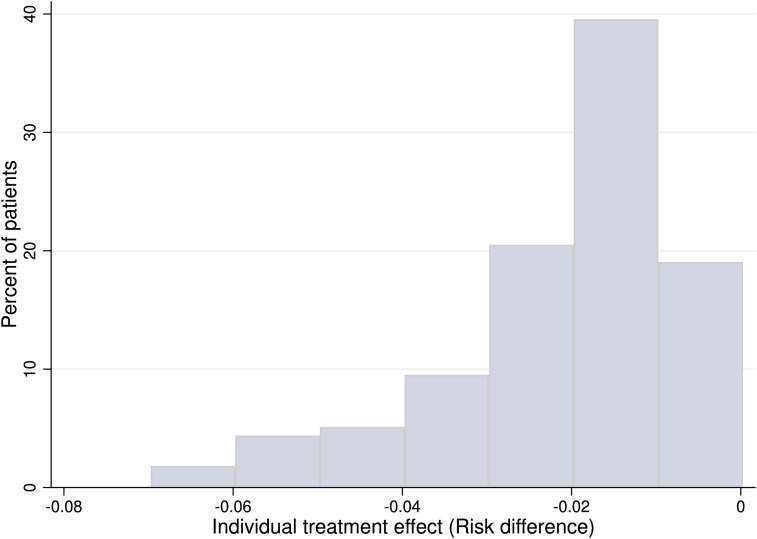
Distribution of individual treatment effect of primary wound closure versus delayed primary wound closure.

The counterfactual prediction model estimated the probability of SSI for receiving the assigned intervention (factual) or an alternative intervention (counterfactual) given participant characteristics based on type of appendicitis, diabetes, fecal contamination, and incision length. Although incision length is a continuous variable, a practical incision length of 2–10 cm was assigned. As a result, an iTE varied from −0.070 to −0.004, representing an absolute reduction in SSI risk between 0.4% and 7% for those in receipt of PC as opposed to DPC (Fig. 2). Also, ∼79% of participants would have > 1% absolute SSI risk reduction following PC. In addition, 38.1% of participants would have an absolute SSI risk reduction by ≥2.1% (ie, ≥ ATE) following PC.

For clinical application, the individualized SSI risks by iTE were estimated from the counterfactual risk prediction model for different predictor subgroups (Table 3). The greatest reduction in risk of SSI (7%) was detected in diabetic participants with ruptured appendicitis and fecal contamination with incision length of 10 cm, corresponding to SSI risks of 47.1% and 54.1% for PC and DPC, respectively. The smallest differences in SSI risk between PC and DPC participants were identified for nondiabetic patients with gangrenous appendicitis, although the iTEs still favored PC. Individualized SSI risks varied between 1% and 3.8% for PC patients and 1.3% to 4.9% for DPC with iTE of 0.3%–1.2%.

**Table 3.** Estimation of Individualized Patient Risk of Surgical-Site Infection From Different Wound Closure Strategies: A Counterfactual Prediction Model

Diabetes	Type of Appendicitis	Fecal Contamination	Incision Length, cm	SSI (%)		% Risk Reduction
				PC	DPC	
Yes	Ruptured	Yes	2	18.8	23.5	**4.7**
			5	27.8	33.7	**6**
			7	35	41.6	**6.6**
			10	47.1	54.1	**7**
		No	2	8.5	11	**2.5**
			5	13.4	17	**3.6**
			7	17.8	22.3	**4.5**
			10	26.4	32.2	**5.8**
	Gangrenous	Yes	2	7.8	10.1	**2.3**
			5	12.4	15.7	**3.4**
			7	16.5	20.7	**4.2**
			10	24.6	30.2	**5.6**
		No	2	3.3	4.3	1
			5	5.4	7	1.6
			7	7.4	9.5	**2.2**
			10	11.6	14.8	**3.2**
No	Ruptured	Yes	2	6.5	8.4	1.9
			5	10.3	13.2	**2.9**
			7	13.8	17.5	**3.7**
			10	21	26	**5**
		No	2	2.7	3.6	0.8
			5	4.4	5.7	1.3
			7	6.1	7.9	1.8
			10	9.6	12.4	**2.7**
	Gangrenous	Yes	2	2.5	3.3	0.8
			5	4	5.3	1.2
			7	5.6	7.2	1.7
			10	8.9	11.4	**2.5**
		No	2	1	1.3	0.3
			5	1.7	2.2	0.5
			7	2.3	3	0.7
			10	3.8	4.9	1.2

Note. DPC, delayed primary wound closure; PC, primary wound closure; SSI, surgical site infection. Bolds indicate where individual’s benefit is greater than average.

## Discussion

We conducted a secondary analysis of RCT data using counterfactual modeling to predict individualized patient risk of SSI following wound closure according to appendicitis type, diabetes status, wound contamination, and incision length. Our findings suggest that all patients potentially benefit from reduced SSI risk following PC compared to DPC; in other words, no patient profile favored DPC over PC. Although the predicted potential benefit for some patients was small (0.4%), for others it was as high as 7%, particularly in high-risk patients who were diabetic, had ruptured appendicitis, a contaminated wound, or an incision length of 7–10 cm.

Previous evidence^7–9^ supported the use of DPC in contaminated surgical wounds, which has become the mainstream of surgical practice. However, the most recent guideline^21^ stated that DPC does not reduce SSI risk in contaminated or dirty appendectomy incision, based on the findings of this study and a meta-analysis.^22^ Although the meta-analysis did not detect a significant effect of DPC over PC, the evidence suggested that DPC might be beneficial for patients in developing countries. However, our findings did not support this conclusion.

Of the 6 RCTs^10,23–27^ published between 1981 and 2012 that were included in our previously published systematic review,^3^ 4 RCTs^10,23–25^ reported lower SSI risk following PC compared to DPC, but only 1 RCT reported this difference to be significant.^10^ The number of patients included in these RCTs was small, ranging between 44 and 122 participants. Although our RCT^12^ had the largest sample size reported to date with 607 patients, SSI risk following PC did not differ significantly to that of DPC. However, treatment response is variable and potentially influenced by patient characteristics and intraoperative variation. Therefore, the need for a counterfactual prediction model to estimate SSI risk at an individual patient level was warranted.

Counterfactual prediction modeling has been recently applied in other clinical areas such as therapeutic studies.^28–34^ Treatment plan customization for individual patients can be achieved by comparison of iTE with adverse event risk.^30^ Furthermore, more meaningful clinical interpretation is provided by the conversion of iTE to individual number needed to treat (iNNT),^30^ which reflects the absolute inverse iTE value. Unfortunately, as far as we are aware, surgical studies have yet to consider a counterfactual prediction modeling approach for the estimation of iTEs.

Nevertheless, the validity of the iTE estimate is dependent on the validity of the assumptions that underpin the counterfactual prediction model.^30^ Our study provided a robust counterfactual prediction model given the high level of discrimination and calibration. Although our RCT included many participants with complicated appendicitis, the number of SSI events was relatively low and was restricted to 48 of the 546 patients included in the analysis. As a rule of thumb, the inclusion of 5 events per predictor variable in the counterfactual model should improve its validity and reduce overfitting as indicated by our calibration performance.

Some operative factors that were previously associated with SSI were not included in this counterfactual prediction model for the following reasons. Most patients received antibiotics (either third-generation of cephalosporine, metronidazole, or ciprofloxacin), and antibiotic use was not significantly associated with SSI occurrence; thus, it was not included in a counterfactual prediction model. Likewise, all patients received intraoperative lavage, and the lavage technique was standardized. Postoperative factors (eg, operation time,^18^ use of staples,^35^ and use of antimicrobial dressing^36^) were not considered in our model because we collected data regarding preoperative or intraoperative factors that occurred before wound closure in order to guide the selection of wound-closure procedure. Obviously, postoperative factors were not available at the time of clinical decision making.

To the best of our knowledge, no other study has implemented a counterfactual prediction model to estimate iTE following general surgery using data from an RCT, in this case using complicated appendectomy as an example. Our findings highlight the significant reduction in SSI offered through estimated iTE associated with PC compared to DPC. Our findings help simplify the wound-closure decision for clinicians and individual patients. All patient profiles show better outcomes with PC rather than DPC, but the magnitude of this benefit varied across patient profiles.

This study had several limitations. First, although we used data from many complicated appendicitis patients, the number of recorded SSI events was low, limiting the predictive ability of the variables included. Second, we only included patients that had all covariate data available, which accounted for 91% of the cohort. Although we did not perform imputation for missing data, we would anticipate that any bias that might result would be minimal given that only 9% of participants were excluded. Third, a few important factors were not considered (eg, severity of diabetes and amount of wound contamination) due to lack of data. Fourth, only open appendectomy was performed according to the original trial protocol. The use of our prediction model in laparoscopic approach should be carefully considered if the SSI rate differs significantly from that reported in our study. Fifth, outcome assessors could not be blinded given that superficial SSI ascertainment was a subjective assessment. Ascertainment bias might be present. However, diagnosis of SSI strictly followed the CDC criteria,^19^ which should minimize ascertainment bias. Finally, we have yet to externally validate our counterfactual prediction model in other settings; thus, generalization of our findings should be applied with care, particularly where the SSI rate is higher or lower than that in our setting (ie, 8.8%). Further prospective study should be conducted to evaluate the clinical impact of our model in other settings with various SSI and deep and organ-space infection rates.

In conclusion, PC may be the preferred treatment option for appendectomy wound closure leading to reduced SSI risk based on iTE, although the magnitude of the absolute benefit varies according to clinical characteristics. In particular, the benefits of PC were more obvious in high-risk patients with diabetes, ruptured appendicitis, contaminated wound, and long incision. This counterfactual model will guide surgeons and patients in shared decision making for appendectomy wound management.

## Supplementary material

For supplementary material accompanying this paper visit https://doi.org/10.1017/ice.2023.214. click here to view supplementary material
