# Supplementary material for: Delayed versus primary closure to minimize risk of surgical-site infection for complicated appendicitis: A secondary analysis of a randomized trial using counterfactual prediction modeling
Source: Infect Control Hosp Epidemiol. 2023 Nov 6;45(3):322–8. doi: 10.1017/ice.2023.214 (PMC10933508; doi:10.1017/ice.2023.214)
Supplement: Supplementary file 1 [file S0899823X23002143sup001.docx]

**Supplementary Materials**

**Delayed versus Primary Closure to Minimize Risk of Surgical Site Infection for Complicated Appendicitis: A Secondary Analysis of a Randomized Trial Using Counterfactual Prediction Modeling**

Amarit Tansawet^1,2^; Boonying Siribumrungwong^3,*^; Suphakarn Techapongsatorn^1^; Pawin Numthavaj^2^; Napaphat Poprom^4^; Gareth McKay^5^; John Attia^6^; Ammarin Thakkinstian^2,*^

^1^ Department of Surgery, Faculty of Medicine Vajira Hospital, Navamindradhiraj University, Bangkok, Thailand

^2^ Department of Clinical Epidemiology and Biostatistics, Faculty of Medicine Ramathibodi Hospital, Mahidol University, Bangkok, Thailand

^3^ Department of Surgery, Faculty of Medicine, Thammasat University, Pathum Thani, Thailand

^4^ Department of Surgery, Faculty of Medicine Ramathibodi Hospital, Mahidol University, Bangkok, Thailand

^5^ Centre for Public Health, School of Medicine, Dentistry and Biomedical Sciences, Queen’s University Belfast, Belfast, UK

^6^ School of Medicine and Public Health, and Hunter Medical Research Institute, University of Newcastle, New Lambton, New South Wales, Australia

^*^ Equal contribution

**Corresponding author**

**Ammarin Thakkinstian,** PhD. Department of Clinical Epidemiology and Biostatistics, Faculty of Medicine Ramathibodi Hospital, Mahidol University, 4^th^ Floor Sukho Place Building, 218/11 Sukhothai Road, Dusit, Bangkok 10300, Thailand.

Tel: +66-2-201-1877, Fax: +66-2-201-1284, Email: [ammarin.tha@mahidol.ac.th](mailto:ammarin.tha@mahidol.ac.th)

**Table S1.** Patient-related summary characteristics and intraoperative data by wound closure approach (page 2)

**Fig. S1.** Surgical site infection prediction model calibration plot (page 3)

**Table S1.** Patient-related summary characteristics and intraoperative data by would closure

| **Patient-related factors** | Total  (n = 546) | PC  (n = 271) | DPC  (n = 275) |
| --- | --- | --- | --- |
| Age, year, mean (SD) | 45.6 (18.2) | 45.7 (18.3) | 45.6 (18.2) |
| Sex, male (%) | 292 (53.5) | 149 (55) | 143 (52) |
| BMI, kg/m^2^, mean (SD) | 23.5 (4.4) | 23.5 (4.4) | 23.4 (4.3) |
| Smoking (%) | 85 (15.6) | 44 (16.2) | 41 (14.9) |
| ASA classification (%) |  |  |  |
| Class I, II | 468 (86.4) | 227 (84.4) | 241 (88.3) |
| Class III, IV | 74 (13.7) | 42 (15.6) | 32 (11.7) |
| Diabetes (%) | 49 (9) | 20 (7.4) | 29 (10.6) |
| Hypertension (%) | 108 (19.8) | 57 (21) | 51 (18.6) |
| Symptom duration, hours, median (IQR) | 24 (15, 48) | 24 (15, 48) | 24 (17, 48) |
| Presence of fever (%) | 303 (55.6) | 152 (55.3) | 151 (55.9) |
| WBC count, cell/mm^3^, mean (SD) | 15 752 (5013) | 15 894 (5014) | 15 613 (5017) |
| Hematocrit, %, mean (SD) | 39.1 (6.5) | 39.3 (6.5) | 38.9 (6.5) |
| **Pre-Intraoperative factors** |  |  |  |
| Pre-operative antibiotic use (%) |  |  |  |
| Yes | 524 (96) | 260 (95.9) | 264 (96) |
| No | 22 (4) | 11 (4.1) | 11 (4) |
| Type of appendicitis (%) |  |  |  |
| Gangrenous | 128 (23.4) | 61 (22.5) | 67 (24.4) |
| Ruptured | 418 (76.6) | 210 (77.5) | 208 (75.6) |
| Fecal contamination (%) | 161 (29.5) | 78 (28.8) | 83 (30.2) |
| Incision length, cm,  mean (SD) | 5.8 (2.3) | 5.8 (2.4) | 5.8 (2.3) |
| Subcutaneous tissue thickness, cm, median (IQR) | 2.8 (1.5, 4.6) | 2.8 (1.5, 4.7) | 2.5 (1.5, 4.6) |
| Suction drain use (%) | 109 (20) | 52 (19.2) | 57 (20.8) |
| Operation time, minute, mean (SD) | 51 (42.5) | 52.8 (44.8) | 49.2 (40) |
| **Outcome** |  |  |  |
| SSI (%) | 48 (8.8) | 20 (7.4) | 28 (10.2) |

ASA, American Society of Anesthesiologists; BMI, body mass index; DPC, delayed primary wound closure; IQR, interquartile range; PC, primary wound closure; SD, standard deviation; SSI, surgical site infection; WBC, white blood cell

**Fig. S1.** Surgical site infection prediction model calibration plot
